# The Distribution of Selected Toxic Elements in Sauced Chicken during Their Feeding, Processing, and Storage Stages

**DOI:** 10.3390/foods12071404

**Published:** 2023-03-26

**Authors:** Hangyan Ji, Yuan Zhang, Jianwei Zhao, Xing Zhou, Chenchen Wang, Zhengyu Jin

**Affiliations:** 1 School of Food Science and Technology, Jiangnan University, Wuxi 214122, China; 2 State Key Laboratory of Food Science and Technology, Jiangnan University, Wuxi 214122, China; 3 Collaborative Innovation Center of Food Safety and Quality Control, Jiangnan University, Wuxi 214122, China

**Keywords:** sauced chicken, toxic elements, distribution discipline, whole industry chains

## Abstract

Sauced chicken is popular food worldwide. However, the elemental pollution of poultry industrialization has led to an increasing health risk concern. In this study, four typical toxic elements, including chromium (Cr), arsenic (As), lead (Pb), and cadmium (Cd), were selected and detected in whole industry chains of sauced chicken preparation by inductively coupled plasma-mass spectrometry. The detection method was optimized and verified with an average recovery of 93.96% to 107.0%. Cr has the highest proportion among the elements during the three stages, while the content of Cd was the least. In the feeding stages, elements were at the highest level in the starter broiler, and the grower broiler was considered to have a good metabolic capacity of them. In addition, the elements were mainly distributed in the chicken kidney, gizzard, liver, leg, wing, and lung. In the processing stage, the elements continued to accumulate from the scalding to the sterilization process. The elements were mainly distributed in the chicken wing, leg, head, and breast. In the storage stage, the elements almost kept constant in the polyamide and polyethylene packaging, while it showed irregular small-range fluctuations in the other two packages. This study provides beneficial references for the toxic element risk management in the whole industry chain.

## 1. Introduction

Chicken meat is one of the most important animal source foods all over the world. By the year 2021, global broiler production reached 99.9 million tons with a growth rate of 0.85%. The broiler production outputs of the four major broiler-producing countries (regions), China, the United States of America, Brazil, and the European Union, have been up to 20.38, 14.7, 14.5, and 10.85 million tons, respectively [1,2]. Sauced chicken is one of the most popular traditional cooked meat products. The marinating processing contributes to an extended shelf life of chicken products, which also provides favorable texture and taste for the raw chicken through the addition of ingredients such as sodium chloride, phosphates, vinegar, sugars, and spices [3,4,5]. Currently, there are more than 100 marinated chicken products on the market in the Chinese provinces of Anhui, Shandong, Liaoning, and Henan. With the acceleration of large-scale and industrialization production of sauced chicken, the problems that exist in the whole industry chain (especially food contamination) have brought many challenges. It is not only necessary to ensure the food safety of raw materials for sauced chicken, but also the commitment to food safety in the processing and marketing of sauced chicken products is critical [6,7].

Toxic element contamination of poultry has long been a worldwide concern, as it generally leads to a potential threat to human health [8,9,10]. The typical toxic elements mainly include mercury (Hg), cadmium (Cd), arsenic (As), lead (Pb), and chromium (Cr) which could originate from atmospheric deposition, industrial emissions, agricultural inputs, or parent materials, respectively [11,12]. The Cd is easy to accumulate in the human body after long-term exposure, which may cause renal tubular injury, cartilage disease, spontaneous fracture, and other diseases [13]. The toxicity of As is different depending on its forms, and arsenite is the most toxic. As poisoning may cause skin lesions and disorders of the nervous system, digestive system, and cardiovascular system [14]. Pb ingestion via livestock meat consumption could pose major threats to human health, and its effects are generally devastating, including anemia, cognitive deficits, and hallucinations, and can be carcinogenic to humans [15]. Cr is a potentially toxic heavy metal, especially the hexavalent compounds, which has risks ranging from skin irritation to cancer development for human health, depending on exposure level, dose, and duration [16]. According to various pieces of legislation, the maximum permissible level of Cd, As, and Pb in poultry meat is generally less than 0.05 mg kg^−1^, 0.5 mg kg^−1^, and 0.1 mg kg^−1^, while for Cr, it is generally less than 1.0 mg kg^−1^ in Europe or China (GB 2762-2017, (EC) No 1881/2006) [17,18].

The elemental contamination could occur in the whole industry chain for sauced chicken products. For sauced chicken production, the upstream of the industrial chain involves various raw materials used for broiler feeding. The contamination source could be the feed, agricultural soils, drinking water, or other risk factors existing in the poultry growth environment, which could occur in the whole industry chain [1,19,20]. The middle reaches of the industrial chain are the production and processing stages. The main processes include hair removal, reshaping, frying, halogenation, and sterilization, which could further concentrate toxic element pollution [7]. The downstream of the industrial chain includes product sales and storage. At this stage, sauced chicken has been sterilized and inspected, but it is still necessary to ensure the safety of the storage procedure from the packages. In addition, the toxic element could distribute in the different body parts of poultry. Yabe et al. [21] found that high content of Pb was in the chicken kidney and liver of a farm near a lead-zinc mine in the Kabwe region of Zambia, while Shah et al. [22] showed that Hg was concentrated in the chicken liver, leg, and breast in poultry farms in Hyderabad, Pakistan. Nevertheless, there are few systematic studies about the toxic element distribution in the whole industry chain of sauced chicken products. In addition, the quantitative content fluctuation and distribution discipline of the specific chicken body parts are also still unclear. Therefore, it is necessary to investigate the occurrence of these elements in the whole industry chain of sauced chicken products so as to identify the point at the industrial production chain where potential risks of elemental contamination occur.

Toxic elements are common food pollutants studied mostly in food safety-related fields, which are easy to accumulate in organisms through the food chain [23,24]. They are also key pollutants affecting the sauced chicken safety. In this study, we have collected the samples during the feeding, processing, and storage stages of sauced chicken in the whole industry chain. Typical toxic elements, including Pb, Cd, Cr, and As in sauced chicken, were selected as the research objects. The distribution discipline of them in the collected stages and the various chicken body parts were systematically studied.

## 2. Materials and Methods

### 2.1. Materials and Reagents

The toxic element standard solution, including Pb, Cd, Cr, and As (1000 mg mL^−1^, solvent: 1.0 mol L^−1^ HNO_3_), was purchased from ANPEL Laboratory Technologies (Shanghai, Co., Ltd., Shanghai, China). The toxic element internal standard solutions, including germanium (Ge), indium (In), and bismuth (Bi) (1000 mg mL^−1^, solvent: 1.0 mol L^−1^ HNO_3_), were provided by the National Center of Analysis and Testing for Nonferrous Metals and Electronic Materials. Acetone and HNO_3_ of guaranteed reagent grade were purchased from Sinopharm Reagent Chemical Co., Ltd. (Shanghai, China). The chickens for method validation were purchased from Auchan supermarket. The chickens in the feeding stage were provided by Anhui Lu’an Ruidehao Feeding Co., Ltd. (Lu’An, China). The chickens in the processing and storage stage were provided by Huixiangyuan Food Co., Ltd. (Guangzhou, China). The chickens used were all the local Ma chickens from Anhui Province. The tools and containers for toxic elements were soaked in HNO_3_ solution with a mass fraction of 10% overnight, washed and dried with ultrapure water.

### 2.2. Preparation of Stock and Working Standard Solutions

The toxic element standard solution (Pb, Cd, Cr, As, 1000 mg mL^−1^) was diluted by 5% HNO_3_ solution to a final concentration of 10 mg L^−1^ in a 200 mL volumetric flask. The prepared solution was used as toxic element stock standard solution and was stored at 4 ℃ in a dark place. The standard stock solution was warmed up to room temperature before use. The mixed standard solution was prepared by diluting 2 mL of various toxic element standard stock solution by acetone to a final concentration of 1000 μg L^−1^ in a 20 mL volumetric flask. The mixed standard solution was then diluted with 5% HNO_3_ solution to obtain a final concentration of 1 μg L^−1^, 5 μg L^−1^, 10 μg L^−1^, 30 μg L^−1^, and 50 μg L^−1^. The prepared solution was used as a toxic element working standard solution. The toxic element internal stock and working standard solutions were prepared as the same method described above.

### 2.3. Sauced Chicken Samples Collection 

#### 2.3.1. The Collection of Sauced Chicken Samples in the Feeding Stage

**Figure 1:**
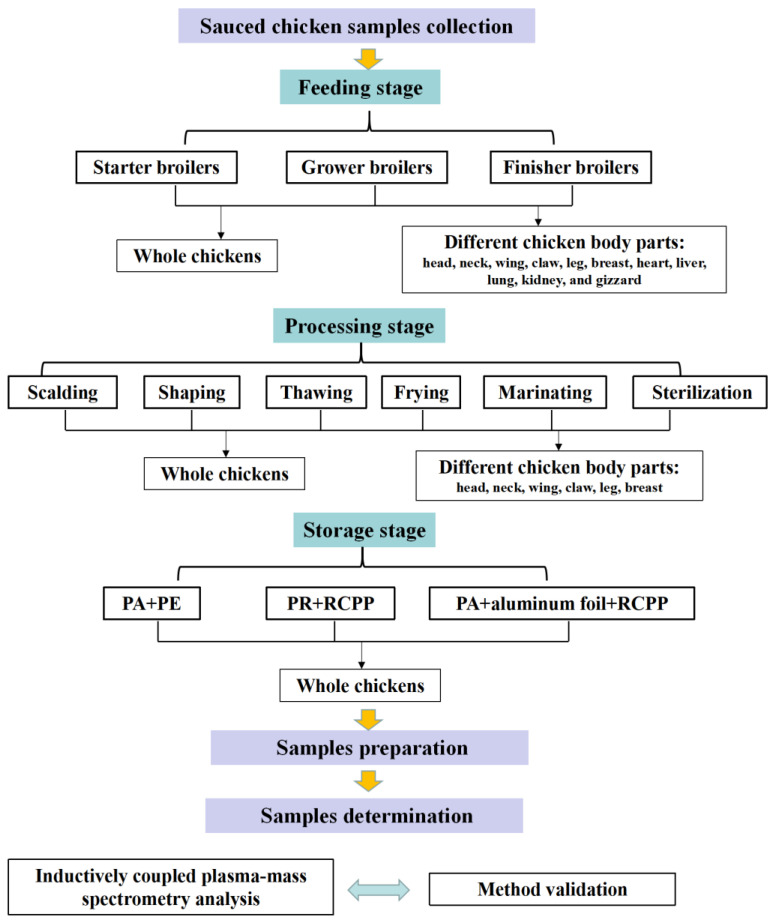
Skeletal scheme of the research methods.

The skeletal schemes of the research methods in this study are indicated in Figure 1. Acquisition and pretreatment of chicken samples were performed according to the approved guidelines [25,26]. The chickens in the feeding stage included starter broilers, grower broilers, and finisher broilers. The starter broilers, grower broilers, and finisher broilers represented chickens fed for one month, two months, and four months, respectively. The whole chickens in the feeding stage were obtained as per the following procedure:

For starter broiler sample preparation, nine of them were randomly selected from the feeding base and were slaughtered, skinned, and boned. Then, the chickens were divided into three groups as parallel determination groups, and then each group was crushed together. Then 0.5 kg of chickens were taken from each group, which were used for toxic element detection. The same treatments were also conducted in the grower broilers and finisher broilers to obtain the broiler samples.

The different body parts of chickens were obtained as per the following procedure: For different body part samples of the starter broiler, nine of them were randomly selected from the feeding base and were slaughtered, skinned, and boned. The body parts were obtained and carefully preserved, including the head, neck, wing, claw, leg, breast, heart, liver, lung, kidney, and gizzard. Each of the body parts was divided into three groups as a parallel determination group, and then each group of chickens was crushed together. Then, 0.05 kg of chicken body parts from each group were used for toxic element detection. The same treatments were also conducted on the grower broilers and finisher broilers to obtain the broiler body part samples.

The above procedures of chicken treatment were all conducted with the help of the related company. All of the chicken samples were put into a white foam box with an ice bag immediately after sampling and stored in the laboratory refrigerator at −20 °C. The pretreatment process of each batch of samples was completed within one week, and the testing process was completed within one month.

#### 2.3.2. The Collection of Sauced Chicken Samples in the Processing Stage

The sauced chicken samples in six main processing stages were selected for toxic element detection, which included scalding, shaping, thawing, frying, marinating, and sterilization. The six processing processes are standardized production procedures of the sauced chicken company, and the chicken samples are representative. The toxic element detection of whole chickens and different body parts of chickens referred to the procedure in the earlier part (Section 2.3.1). The six chicken body parts included the head, neck, wing, claw, leg and breast.

#### 2.3.3. The Collection of Sauced Chicken Samples in Storage Stage

Three different packaging materials were selected for the toxic element detection, which included polyamide and cooking grade cast polypropylene package (PA + RCPP), polyamide and polyethylene package (PA + PE), and polyamide, aluminum foil, and cooking grade cast polypropylene package (PA + aluminum foil + RCPP). The chicken samples were placed at about 30 °C for 20 days in the three packages and tested every five days. The whole chickens were utilized for the toxic element detection referred to in the procedure in the previous Section 2.3.1.

### 2.4. Sample Preparation

The toxic elements were analyzed following the previously reported method with minor modifications [16]. Chicken samples of 1 g were placed in centrifuge tubes of 50 mL added with HNO_3_ of 5 mL. Then the centrifuge tubes were placed in a Heavy Metal Digester (SH230N, Wuxi Keep Apex Technology Co., Ltd., Wuxi, China) at about 120 °C for 40 min. The digested chicken samples were diluted to 50 mL with ultrapure water and then passed through a 0.22 µm inorganic microporous membrane before further instrumental analysis.

### 2.5. Inductively Coupled Plasma-Mass Spectrometry Analysis

The inductively coupled plasma-mass spectrometry (ICP-MS, PerkinElmer, Inc., Waltham, MA, USA) was applied in the four typical toxic element detection as previously described with optimization [27]. The determination was conducted in the following conditions: the element signal strength of Be, In, and U was adjusted to a value above 2000, 40,000, and 30,000, respectively. The background signal value was tuned to lower than or equal to 1. The oxide ion ratio (CeO/Ce) was tuned to lower than or equal to 0.025. The double charge ion ratio (Ce++/Ce) was tuned to lower than or equal to 0.03. The mixed standard solution and internal standard solution of the toxic elements were injected into the ICP-MS, respectively, to obtain the corresponding response value. The standard curve is drawn by taking the ratio of the concentration to be measured and the concentration of the internal standard of the toxic element as abscissa, and the ratio of the response signal value to be measured and the response signal value of the internal standard of the toxic element as ordinate. The content of each toxic element in the sauced chicken sample was calculated as follows:

$$X=\frac{(C\;-{\;C}_{0})\;\times \;V}{M}$$

where X was the toxic element content in the sauced chicken sample (μg kg^−1^), *C* was the mass concentration of the toxic element to be measured in the sample (μg L^−1^), *C*_0_ is the mass concentration of the toxic element to be measured in the blank sample (μg L^−1^), *V* is the volume of the sample solution (mL), *M* is the mass of the sample (g).

### 2.6. Method Validation

The method validation was performed by referring to the previous study with modification [28]. The accuracy, repeatability, and precision have been evaluated for this method with the certified reference of Conformity assessment—Guidance on validation and verification of chemical analytical methods (GB/T 27417-2017). The mixed standard solutions of 50 μL, 250 μL, and 500 μL were added to the sauced chicken sample of 1 g (to the nearest 0.001 g), respectively, to a final spiking concentration of 0.05 mg kg^−1^, 0.250 mg kg^−1^, 0.500 mg kg^−1^. The chicken samples with analyte were placed overnight at room temperature. Each group was prepared with six parallel samples, including a blank group and digested group, according to the method described in Section 2.4. The spiked recovery rate and relative standard deviation were calculated. The recovery rate was calculated as follows:

$$R=\frac{C_{a}-{\;C}_{o}}{C}\;\times \;100\%$$

where *R* was the recovery rate, *C*_a_ was the mass concentration of the toxic element to be tested in the chicken sample after spiking (μg L^−1^), *C*_o_ was the mass concentration of the toxic element to be tested in the blank sample (μg L^−1^), and *C* was the theoretical concentration of the chicken sample after spiking (μg L^−1^).

### 2.7. Statistical Analysis

All results obtained are the average values of three replicates of the samples analyzed at the same time and under the same conditions. Results are expressed as mean ± SD (*n* = 3) with one-way analysis of variance (ANOVA). All experimental data were statistically analyzed using SPSS 23.0 software (SPSS Inc., Chicago, IL, USA). Turkey’s test was used for significant differences (*p* < 0.05).

## 3. Results and Discussion

### 3.1. The Methodological Verification of Toxic Elements

As indicated in Table 1, it can be seen that the correlation coefficients of the regression equation for four toxic elements were all higher than 0.9999 within the linear range of 0.0005~50.0000 μg L^−1^. This result indicated a satisfactory linear relationship for this determination method. The detection limit of Cr, As, Cd, and Pb in chicken samples were 0.0683 μg L^−1^, 0.0607 μg L^−1^, 0.0357 μg L^−1^, and 0.0510 μg L^−1,^ respectively, while the limits of quantitation of the four toxic elements were 0.2277 μg L^−1^, 0.2022 μg L^−1^, 0.1189 μg L^−1^, and 0.1701 μg L^−1^, respectively. The linearity and sensitivity within the operating range of the ICP-MS determination method were adequate for their quantitative analysis in chicken samples. According to methodological verification, as shown in Table 2, the recoveries from the substances spiked with internal standards and analyzed as quality control were close to 100% for the four typical toxic elements. The average recovery rate of Cr, As, Cd, and Pb in chicken samples were 107.0%, 97.45%, 94.35%, and 93.96%, respectively, while the average precision degree of the four toxic elements were 4.933%, 2.937%, 1.927%, and 9.983%, respectively, at three concentration levels. The relative standard deviations (RSD) were all less than 3% at the highest spike level. It can be confirmed that the recovery rate and precision can meet the detection requirements compared to the previous study [29]. This method was with high accuracy and repeatability for the determination of Cr, As, Cd, and Pb in chicken samples.

### 3.2. Toxic Elements Distribution in Chicken in the Feeding Stage

#### 3.2.1. Whole Chicken

**Figure 2:**
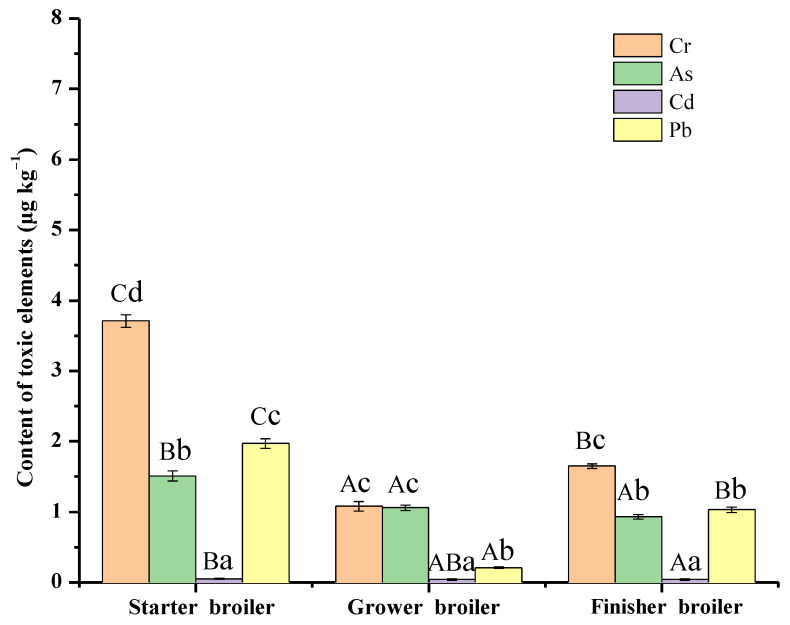
Toxic elements in chicken during the feeding stage. (Capital letters indicate significant differences in the content of the same element in a different feeding stage. Small letters indicate significant differences in the content of different elements in the same feeding stage).

Chicken feeding is usually the key stage involving toxic element pollution, which mainly comes from the daily diet and growth environment [30,31]. The investigation of the fluctuation and distribution discipline of toxic elements in this stage is critical to risk control from the source. The content of the four typical toxic elements in chickens in the feeding stage is indicated in Figure 2. It could be seen that the starter broiler has the highest content of each toxic element. In the finisher broiler, the content of Cr and Pb was higher than it had been in the grower broiler, while the content of As and Cd was close. It could be confirmed that the grower broiler has the least toxic element content. The reasons for this difference in the three feeding stages may include two main aspects: feed intake and metabolic function. For the feed intake, the feed for starter broilers usually was different from the feed for grower and finisher broilers, leading to different total toxic element accumulation in chickens [32]. Therefore, it is worth considering this as the management point in poultry farming to control the toxic elements. For the metabolic function, the organs of starter broilers were generally not fully developed, which had a negative effect on the toxic elements’ metabolism and excretion. Nevertheless, compared to grower broilers, the organs of finisher broilers began to age, and the metabolism function of toxic elements also weakened. Thus, a suitable feeding time could also be important for risk control. As indicated in Figure 2, the highest content of Cr and the least content of Cd are in starter, grower, and finisher broilers. The Cr, As, and Pb were the main toxic elements in chickens, with the Cr and Pb accounting for half of the total toxic element content. It is interesting to notice that the Cr and Pb had similar changing trends in the feeding stage. The As showed a slight downward trend in chickens, while the Cd content (0.04~0.05 μg kg^−1^) in chickens was almost undetectable. This study was new in relation to the current studies and also a favorable supplement to the investigation of the effects of environmental factors on broilers in different feeding stages [33,34].

#### 3.2.2. The Content in Different Body Parts of Chicken

The toxic element distribution in different body parts of chicken is shown in Table 3. For the starter broiler, the Cr was concentrated in the kidney, which was about 10 times higher than it was in other body parts. The As, Cd, and Pb were also at a high level in the starter broiler kidney. This result was similar to the studies confirmed by Hu et al. and He et al., which indicated the kidney was the main organ for heavy metal decrease [30,35]. It could be confirmed that the kidney was the most harmful part to human health in starter broilers. In addition, the broiler claw, heart, leg, and liver had the highest content of Cr, while the broiler wing, breast, lung, and gizzard were rich in As. The head and neck have the highest content of Pb. For the grower broiler, the As and Pb were mainly accumulated in the gizzard, and the Cr and Cd were also at a relatively high level. Thus, the toxic elements could be easily concentrated in the gizzard for the grower broiler. In addition, the broiler wing, breast, heart, kidney, and gizzard have the highest content of Pb, while the head, neck, claw, leg, and lung have the highest content of Cr. Different from the starter boiler, the toxic element distributions were uniform in various body parts of grower broilers at a relatively low level. For finisher broilers, the Cr and As were concentrated in the broiler lung with Cd and Pb also at a high level. Thus, it could be seen that the lung is the most toxic body part for a finisher broiler. Meanwhile, the Cd and Pb were mainly accumulated in the broiler liver and wing, respectively. For other body parts, the broiler head, neck, claw, leg, heart, and gizzard have the highest content of Cr, while the breast and kidney have the highest content of Pb. It could be confirmed that the different body parts tend to accumulate Cr in the three feeding stages. However, there is no obvious distribution regular pattern for the other three toxic elements.

### 3.3. Toxic Elements Distribution in Chicken in the Processing Stage

#### 3.3.1. Whole Chicken

**Figure 3:**
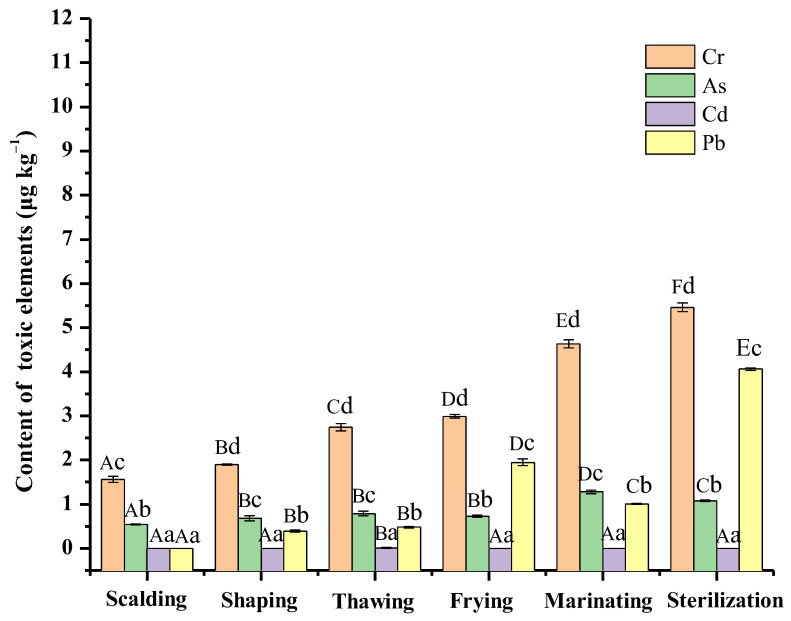
Toxic elements in chicken during the processing stage. (Capital letters indicate significant differences in the content of the same element in the different processing stages. Small letters indicate significant differences in the content of different elements in the same processing stage).

Chicken processing was another important stage for toxic element accumulation [36]. The contents of four typical toxic elements in the processing stage are indicated in Figure 3. Similar to the feeding stage, the Cr also had the highest proportion in the sixth processing stage, and it kept accumulating (from 1.56 μg kg^−1^ to 5.46 μg kg^−1^) during the processing. The As and Pb also almost maintained an increase, while the Cd kept constant during the processing stage. It could be confirmed that the processing stages facilitated the accumulation of toxic element content. For each processing stage, the four toxic elements had regular and varied fluctuation. The Pb significantly increased after the frying treatment. Afterward, it reached the highest content after sterilization treatment. The water loss in the two thermal processing could be an important reason. The Cr and As also obviously increased after the marinating treatment. Chicken marinating has usually been considered a procedure that easily leads to pollution. As previously reported by Lee et al., the amounts of toxic elements transferred from oilseeds, noodles, or tea leaves to boiling water and tea soup increased with the increase of boiling time and tea soup soaking time [37]. This indicated that toxic elements might migrate from the brine soup (9.99 μg kg^−1^ in this experiment) to chicken meat during the processing of chicken with soy sauce. The accumulation of toxic elements in these processes was mainly due to the toxic elements in brine or oil, the addition of other ingredients, and the migration of toxic elements contained in the vessels used as previously reported [38,39]. Thus, the frying process and the utilization of soup stock are critical for risk management. Compared to the other three toxic elements, the Cd content was stable and accounted for the least proportion (within 0.01 μg kg^−1^) in the chicken processing stage.

#### 3.3.2. The Content in Different Body Parts of Chicken

The toxic element distribution in different body parts of chicken in the processing stage is indicated in Table 4. The toxic element distributions were different in various body parts of chicken after each processing procedure. For chicken scalding, Cr and Pb are mainly concentrated in the leg and wing, respectively, while As and Cd have no significant accumulation in different body parts of the chicken. In addition, the chicken head, neck, claw, and breast all had the highest proportion of Cr. For chicken shaping, the Cr and Pb were mainly concentrated in the head and leg, respectively, while the regular distribution of As and Cd was similar to that of chicken scalding. In addition, the Cr also has the highest proportion in the other body parts of the chicken. For chicken thawing, the Cr was mainly concentrated in the chicken wing, and Pb was mainly in the claw, leg, and breast. After chicken frying, the Cr was mainly in the chicken. It is interesting to notice that the content of Pb increased and was uniformly distributed in the six body parts from neck to breast. Similarly, this situation has also been observed for the four toxic elements in various body parts of chicken after the chicken marinating. For chicken sterilization, the Cr and Pb were mainly concentrated in the chicken breast and leg, respectively. In conclusion, Cr mostly has the highest proportion in different body parts of the chicken, and the distribution of As and Cd was relatively uniform within it. Meanwhile, the distribution of Pb was generally irregular in the chicken.

### 3.4. Toxic Elements Distribution in Chicken in Storage Stage

**Figure 4:**
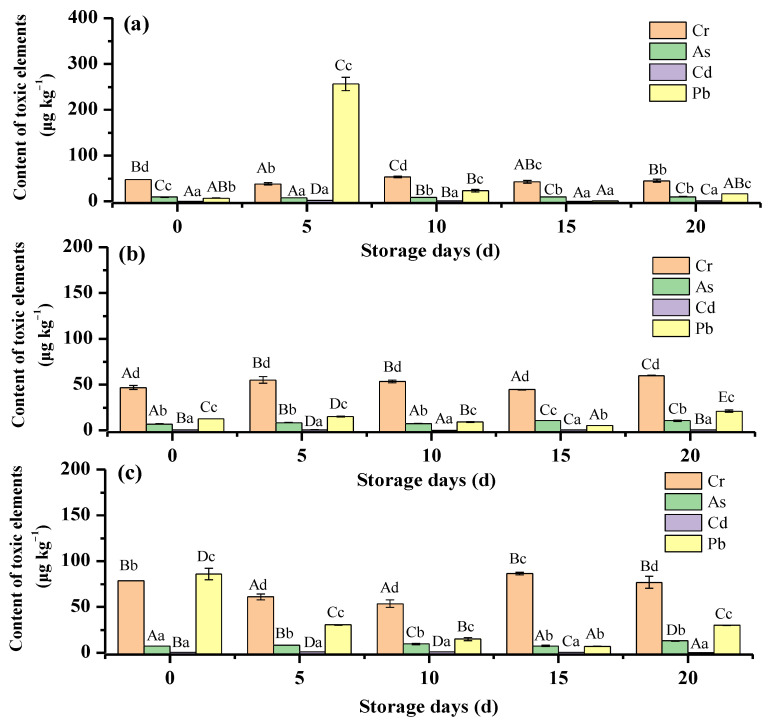
Toxic elements in chicken during storage stage. (**a**) PA + RCPP package, (**b**) PA + PE package, (**c**) PA + aluminum foil + RCPP package. (Capital letters indicate significant differences in the content of the same element in different storage stages. Small letters indicate significant differences in the content of different elements in the same storage stage).

The migration of toxic elements from packaging materials is an important source of chicken contamination [40,41]. The content fluctuation of the four typical toxic elements in the sauced chicken during the storage stage is indicated in Figure 4. It could be noticed the content of the toxic element has a discrepancy with various packaging materials. For the PA + RCPP package, as indicated in Figure 4a, the sauced chicken products had the highest Pb content in the first five days of storage, and the content decreased nearer the initial level for the rest of the fifteen days of storage. The fluctuation of the three other toxic elements is not significant during the 20 days of storage. For the PA + PE package, as indicated in Figure 4b, the content of the four toxic elements kept stable in the storage stage. For the PA + aluminum foil + RCPP package, as indicated in Figure 4c, it was found that this type of package had the highest initial Cr and Pb content. The Cr content decreased in the first 10 days of storage and then slightly increased in the remaining 10 days of storage. For the Pb, the content decreased in the first 15 days of storage and then increased in the remaining 5 days of storage. Among the three various packages, the PA + PE package had the most stable toxic element content during the long storage time. In addition, there was no significant regular fluctuation of the toxic element content for the three packages. However, it is necessary to note that the contents of a toxic element in the three various chicken packages were all under the international standard limit.

## 4. Conclusions

Sauced chicken is a popular food worldwide for their unique flavor and favorable storage characteristics. The toxic element content is a key factor that determines the quality of sauced chicken products. Here, we systematically studied the distribution discipline of elements during the representative chicken feeding, processing, and storage stages in a whole industry chain. Among the four typically selected toxic elements, Cr was the key elemental pollution risk source, followed by As and Pb. The distribution of the elements varied over three feeding stages. It was inferred that the grower broiler could be the suitable raw material for sauced chicken preparation from the perspective of toxic elements content. Each process promoted elemental accumulation. In particular, frying and sterilization significantly increased the Pb content. Thus, the processing stages could all be necessary for elemental risk management. However, the influence of the packages was not obvious during 20 days of storage except for Pb in the PA+RCPP package. It is also worth noting that the toxic elements were mainly distributed in the chicken kidney, gizzard, liver, leg, wing, and lung in the feeding stage, while they were mainly in the wing, leg, head, and breast in the processing stage. These findings are conducive to the discovery of element pollution risk points. In the next step of this research, it is necessary to further conduct the traceability of pollutants in specific points involved in the whole industry chain of sauced chicken preparation.

## Figures and Tables

**Table 1:** Results of methodological verification of toxic elements in chicken.

Toxic Element Types	Internal Standard Element	Regression Equation	Linear Range (μg L^−1^)	Correlation Coefficient	Detection Limit (μg L^−1^)	Limit of Quantitation (μg L^−1^)
Cr	Ge 74	y = 0.083x	0.0005~50.0000	0.9999	0.0683	0.2277
As	Ge 74	y = 0.082x	0.0005~50.0000	0.9999	0.0607	0.2022
Cd	In 115	y = 0.018x	0.0005~50.0000	0.9999	0.0357	0.1189
Pb	Bi 209	y = 0.134x	0.0005~50.0000	0.9999	0.0510	0.1701

**Table 2:** Spiked recovery and relative standard deviation of toxic elements in chicken (*n* = 6).

Toxic Element Types	Added Level (μg L^−1^)	Recovery Rate (%)	Relative Standard Deviation (%)
Cr	1.00	106.0	8.160
	5.00	109.2	3.890
	10.0	105.8	2.750
As	1.00	96.43	3.520
	5.00	98.82	3.110
	10.0	97.11	2.180
Cd	1.00	95.14	2.180
	5.00	94.71	2.140
	10.0	93.20	1.460
Pb	1.00	90.86	15.49
	5.00	99.52	12.33
	10.0	91.50	2.130

**Table 3:** Toxic elements in different body parts of chicken during the feeding stage.

Feeding Stage	Chicken Body Parts	Cr (μg kg^−1^)	As (μg kg^−1^)	Cd (μg kg^−1^)	Pb (μg kg^−1^)
Starter broiler	Head	2.06 ± 0.02 ^Cc^	1.40 ± 0.01 ^Bb^	0.08 ± 0.01 ^ABCa^	6.82 ± 0.02 ^Hd^
	Neck	2.92 ± 0.01 ^Dc^	1.89 ± 0.09 ^Db^	0.13 ± 0.01 ^CDa^	4.10 ± 0.08 ^Fd^
	Wing	3.00 ± 0.02 ^Dc^	3.21 ± 0.03 ^Hd^	0.04 ± 0.01 ^Aa^	0.62 ± 0.04 ^ABb^
	Claw	3.01 ± 0.10 ^Dc^	0.90 ± 0.02 ^Ab^	0.06 ± 0.01 ^ABa^	2.92 ± 0.06 ^Ec^
	Leg	1.96 ± 0.02 ^BCd^	1.70 ± 0.06 ^Cc^	0.04 ± 0.01 ^Aa^	0.51 ± 0.01 ^Ab^
	Breast	1.26 ± 0.10 ^Ab^	2.73 ± 0.01 ^Fc^	0.05 ± 0.01 ^ABa^	1.08 ± 0.11 ^Cb^
	Heart	3.01 ± 0.01 ^Dd^	1.39 ± 0.07 ^Bb^	0.10 ± 0.01 ^BCDa^	1.71 ± 0.08 ^Dc^
	Liver	2.86 ± 0.06 ^Dd^	2.40 ± 0.05 ^Ec^	1.77 ± 0.04 ^Gb^	1.14 ± 0.10 ^Ca^
	Lung	1.87 ± 0.08 ^Bc^	2.77 ± 0.02 ^Fd^	0.14 ± 0.01 ^Da^	1.74 ± 0.04 ^Db^
	Kidney	20.59 ± 0.10 ^Ed^	2.94 ± 0.03 ^Gb^	0.41 ± 0.01 ^Ea^	5.96 ± 0.02 ^Gc^
	Gizzard	1.97 ± 0.07 ^BCc^	5.00 ± 0.03 ^Id^	0.52 ± 0.05 ^Fa^	0.68 ± 0.05 ^Bb^
Grower broiler	Head	2.07 ± 0.04 ^Ed^	0.61 ± 0.05 ^Bc^	0.03 ± 0.01 ^Aa^	0.43 ± 0.01 ^Bb^
	Neck	1.83 ± 0.06 ^CDd^	0.88 ± 0.03 ^Cb^	0.05 ± 0.01 ^ABa^	1.42 ± 0.05 ^Dc^
	Wing	1.85 ± 0.02 ^CDc^	1.06 ± 0.08 ^Db^	0.02 ± 0.01 ^Aa^	1.97 ± 0.07 ^Ec^
	Claw	3.03 ± 0.05 ^Fd^	0.38 ± 0.02 ^Ab^	0.06 ± 0.01 ^ABa^	2.67 ± 0.02 ^Fc^
	Leg	4.31 ± 0.05 ^Gc^	1.05 ± 0.03 ^Db^	0.05 ± 0.01 ^ABa^	0.06 ± 0.01 ^Aa^
	Breast	1.69 ± 0.11 ^BCc^	1.04 ± 0.04 ^Db^	0.03 ± 0.01 ^Aa^	2.57 ± 0.04 ^Fd^
	Heart	1.83 ± 0.08 ^CDc^	0.68 ± 0.03 ^Bb^	0.05 ± 0.01 ^ABa^	2.95 ± 0.03 ^Gd^
	Liver	0.96 ± 0.04 ^Ab^	0.80 ± 0.04 ^Ca^	1.62 ± 0.06 ^Ed^	1.20 ± 0.09 ^Cc^
	Lung	1.58 ± 0.08 ^Bd^	1.07 ± 0.07 ^Dc^	0.13 ± 0.01 ^Ca^	0.52 ± 0.04 ^Bb^
	Kidney	2.88 ± 0.07 ^Fc^	0.62 ± 0.04 ^Bb^	0.10 ± 0.01 ^BCa^	4.84 ± 0.07 ^Hd^
	Gizzard	1.96 ± 0.07 ^DEb^	2.44 ± 0.08 ^Ec^	0.25 ± 0.01 ^Da^	6.59 ± 0.02 ^Id^
Finisher broiler	Head	5.22 ± 0.10 ^Fd^	0.79 ± 0.01 ^Db^	0.12 ± 0.01 ^Da^	3.79 ± 0.02 ^Gc^
	Neck	1.42 ± 0.05 ^Bd^	0.79 ± 0.04 ^Dc^	0.09 ± 0.01 ^Ca^	0.38 ± 0.02 ^Bb^
	Wing	1.65 ± 0.02 ^Cc^	0.99 ± 0.10 ^Eb^	0.03 ± 0.01 ^Ba^	4.02 ± 0.06 ^Hd^
	Claw	3.98 ± 0.10 ^Ed^	0.39 ± 0.01 ^Ab^	0.09 ± 0.01 ^Ca^	0.90 ± 0.03 ^Dc^
	Leg	1.38 ± 0.04 ^Bc^	0.73 ± 0.01 ^CDb^	<LOQ	0.04 ± 0.01 ^Aa^
	Breast	1.15 ± 0.01 ^Ac^	0.67 ± 0.01 ^Cb^	<LOQ	2.09 ± 0.12 ^Ed^
	Heart	1.60 ± 0.07 ^Cc^	0.96 ± 0.03 ^Eb^	<LOQ	0.02 ± 0.01 ^Aa^
	Liver	1.67 ± 0.02 ^Cc^	0.54 ± 0.03 ^Ba^	4.45 ± 0.01 ^Gd^	0.62 ± 0.02 ^Cb^
	Lung	6.32 ± 0.04 ^Gd^	1.72 ± 0.03 ^Gb^	0.26 ± 0.01 ^Ea^	3.53 ± 0.03 ^Fc^
	Kidney	1.61 ± 0.00 ^Cc^	0.51 ± 0.01 ^Bb^	0.00 ^Aa^	2.15 ± 0.07 ^Ed^
	Gizzard	3.39 ± 0.02 ^Dd^	1.58 ± 0.04 ^Fc^	0.33 ± 0.01 ^Fa^	0.49 ± 0.02 ^Bb^

Capital letters indicate significant differences in the content of the same element in different body parts of the chicken. Small letters indicate significant differences in the content of different elements in the same body parts of the chicken.

**Table 4:** Toxic elements in different body parts of chicken during the processing stage.

Processing Stage	Chicken Body Parts	Cr (μg kg^−1^)	As (μg kg^−1^)	Cd (μg kg^−1^)	Pb (μg kg^−1^)
Chicken scalding	Head	1.91 ± 0.02 ^BCd^	0.34 ± 0.01 ^CDb^	0.05 ± 0.01 ^Ba^	0.55 ± 0.04 ^Cc^
	Neck	1.76 ± 0.04 ^Bc^	0.23 ± 0.01 ^Ab^	0.03 ± 0.01 ^ABa^	0.29 ± 0.02 ^ABb^
	Wing	2.06 ± 0.08 ^Cd^	0.31 ± 0.03 ^BCb^	0.02 ± 0.01 ^Aa^	1.15 ± 0.08 ^Dc^
	Claw	2.05 ± 0.05 ^Cd^	0.25 ± 0.01 ^ABb^	0.02 ± 0.01 ^Aa^	0.34 ± 0.01 ^Bc^
	Leg	2.80 ± 0.09 ^Dd^	0.39 ± 0.02 ^Dc^	0.03 ± 0.01 ^ABa^	0.25 ± 0.01 ^ABb^
	Breast	1.27 ± 0.05 ^Ad^	0.33 ± 0.04 ^CDc^	0.03 ± 0.01 ^ABa^	0.20 ± 0.02 ^Ab^
Chicken shaping	Head	2.88 ± 0.03 ^Ed^	0.22 ± 0.02 ^Bb^	0.03 ± 0.01 ^Aa^	0.40 ± 0.02 ^CDc^
	Neck	5.13 ± 0.09 ^Fc^	0.32 ± 0.01 ^Cb^	0.03 ± 0.01 ^Aa^	0.31 ± 0.02 ^BCb^
	Wing	1.30 ± 0.05 ^Cd^	0.37 ± 0.02 ^Cb^	0.03 ± 0.01 ^Aa^	0.45 ± 0.01 ^Dc^
	Claw	2.63 ± 0.07 ^Dc^	0.12 ± 0.01 ^Aa^	0.03 ± 0.01 ^Aa^	0.28 ± 0.01 ^Bb^
	Leg	0.79 ± 0.01 ^Ac^	0.36 ± 0.01 ^Cb^	0.02 ± 0.01 ^Aa^	0.88 ± 0.09 ^Ec^
	Breast	0.95 ± 0.05 ^Bc^	0.35 ± 0.03 ^Cb^	0.02 ± 0.01 ^Aa^	0.09 ± 0.01 ^Aa^
Chicken thawing	Head	2.35 ± 0.10 ^Dc^	0.27 ± 0.01 ^Ab^	0.01 ± 0.01 ^Aa^	0.35 ± 0.04 ^Ab^
	Neck	1.24 ± 0.07 ^Ad^	0.33 ± 0.01 ^Bb^	0.01 ± 0.01 ^Aa^	0.43 ± 0.02 ^ABc^
	Wing	7.59 ± 0.04 ^Ec^	0.48 ± 0.02 ^Db^	0.02 ± 0.01 ^ABa^	0.51 ± 0.05 ^Bb^
	Claw	1.98 ± 0.07 ^Cc^	0.24 ± 0.01 ^Ab^	0.04 ± 0.01 ^BCa^	6.05 ± 0.08 ^Dd^
	Leg	1.54 ± 0.09 ^Bc^	0.41 ± 0.03 ^Cb^	0.05 ± 0.01 ^Ca^	5.14 ± 0.01 ^Cd^
	Breast	1.11 ± 0.09 ^Ac^	0.48 ± 0.01 ^Db^	0.06 ± 0.01 ^Ca^	6.17 ± 0.09 ^Dd^
Chicken frying	Head	4.99 ± 0.09 ^Ec^	0.29 ± 0.01 ^Ab^	0.02 ± 0.01 ^Aa^	5.46 ± 0.08 ^Dd^
	Neck	1.51 ± 0.01 ^Ac^	0.37 ± 0.01 ^ABb^	0.03 ± 0.01 ^Aa^	3.39 ± 0.05 ^Ad^
	Wing	1.89 ± 0.07 ^Bc^	0.48 ± 0.06 ^Cb^	0.03 ± 0.01 ^Aa^	3.52 ± 0.08 ^ABd^
	Claw	4.32 ± 0.04 ^Dd^	0.29 ± 0.01 ^Ab^	0.03 ± 0.01 ^Aa^	3.60 ± 0.06 ^BCc^
	Leg	1.50 ± 0.05 ^Ac^	0.44 ± 0.03 ^BCb^	0.02 ± 0.01 ^Aa^	3.76 ± 0.11 ^Cd^
	Breast	2.24 ± 0.10 ^Cc^	0.43 ± 0.02 ^BCb^	0.03 ± 0.01 ^Aa^	3.39 ± 0.04 ^Ad^
Chicken marinating	Head	6.75 ± 0.05 ^Dd^	0.65 ± 0.02 ^Ac^	0.07 ± 0.01 ^Da^	0.26 ± 0.03 ^Bb^
	Neck	6.02 ± 0.05 ^Cc^	0.96 ± 0.02 ^BCb^	0.06 ± 0.01 ^CDa^	0.04 ± 0.01 ^Aa^
	Wing	5.26 ± 0.07 ^Bd^	1.04 ± 0.03 ^Cb^	0.04 ± 0.01 ^BCa^	1.55 ± 0.05 ^Dc^
	Claw	6.98 ± 0.21 ^Dc^	0.58 ± 0.01 ^Ab^	0.08 ± 0.01 ^Da^	0.84 ± 0.01 ^Cc^
	Leg	8.73 ± 0.08 ^Ed^	0.88 ± 0.05 ^Bb^	0.02 ± 0.01 ^ABa^	1.48 ± 0.06 ^Dc^
	Breast	4.97 ± 0.08 ^Ac^	0.98 ± 0.06 ^Cb^	0.01 ± 0.01 ^Aa^	0.91 ± 0.10 ^Cb^
Chicken sterilization	Head	5.06 ± 0.09 ^Bd^	0.90 ± 0.04 ^Bc^	0.10 ± 0.01 ^Ca^	0.72 ± 0.01 ^Bb^
	Neck	5.40 ± 0.03 ^Cd^	0.90 ± 0.01 ^Bb^	0.06 ± 0.01 ^ABa^	1.42 ± 0.10 ^Dc^
	Wing	4.65 ± 0.09 ^Ad^	1.07 ± 0.07 ^CDc^	0.07 ± 0.01 ^Ba^	0.88 ± 0.02 ^Cb^
	Claw	6.52 ± 0.05 ^Dd^	0.58 ± 0.04 ^Ac^	0.10 ± 0.01 ^Ca^	0.22 ± 0.02 ^Ab^
	Leg	5.54 ± 0.09 ^Cd^	1.01 ± 0.06 ^BCb^	0.06 ± 0.01 ^ABa^	2.13 ± 0.04 ^Ec^
	Breast	10.62 ± 0.05 ^Ed^	1.19 ± 0.04 ^Dc^	0.04 ± 0.01 ^Aa^	0.66 ± 0.02 ^Bb^

Capital letters indicate significant differences in the content of the same element in different body parts of the chicken. Small letters indicate significant differences in the content of different elements in the same body parts of the chicken.

## Data Availability

Data is contained within the article.

## References

[1] Guo W., Pan B., Sakkiah S., Yavas G., Ge W., Zou W., Tong W., Hong H. (2019). Persistent organic pollutants in food: Contamination sources, health effects and detection methods. Int. J. Environ. Res. Public Health.

[2] Wen X., Li L., Sun S., He Q., Tsai F.-S. (2019). The contribution of chicken products’ export to economic growth: Evidence from China, the United States, and Brazil. Sustainability.

[3] Li Y., Feng T., Sun J., Guo L., Wang B., Huang M., Xu X., Yu J., Ho H. (2020). Physicochemical and microstructural attributes of marinated chicken breast influenced by breathing ultrasonic tumbling. Ultrason. Sonochem..

[4] Lytou A., Panagou E., Nychas G.-J. (2017). Effect of different marinating conditions on the evolution of spoilage microbiota and metabolomic profile of chicken breast fillets. Food Microbiol..

[5] Karam L., Roustom R., Abiad M., El-Obeid T., Savvaidis I. (2019). Combined effects of thymol, carvacrol and packaging on the shelf-life of marinated chicken. Int. J. Food Microbiol..

[6] Garvey M. (2019). Food pollution: A comprehensive review of chemical and biological sources of food contamination and impact on human health. Nutrire.

[7] Li C., Li C., Yu H., Cheng Y., Xie Y., Yao W., Guo Y., Qian H. (2021). Chemical food contaminants during food processing: Sources and control. Crit. Rev. Food Sci. Nutr..

[8] Wan Y., Huang Q., Wang Q., Yu Y., Su D., Qiao Y., Li H. (2020). Accumulation and bioavailability of heavy metals in an acid soil and their uptake by paddy rice under continuous application of chicken and swine manure. J. Hazard. Mater..

[9] Hussien H., Abd-Elmegied A., Ghareeb D., Hafez H., Ahmed H., El-moneam N. (2018). Neuroprotective effect of berberine against environmental heavy metals-induced neurotoxicity and Alzheimer’s-like disease in rats. Food Chem. Toxicol..

[10] Kapaj S., Peterson H., Liber K., Bhattacharya P. (2006). Human health effects from chronic arsenic poisoning-a review. J. Environ. Sci. Health Part A.

[11] Gan Y., Huang X., Li S., Liu N., Li Y., Freidenreich A., Wang W., Wang R., Dai J. (2019). Source quantification and potential risk of mercury, cadmium, arsenic, lead, and chromium in farmland soils of Yellow River Delta. J. Clean. Prod..

[12] Yang L., Wu P., Yang W. (2022). Characteristics, health risk assessment, and transfer model of heavy metals in the soil-food chain in cultivated land in Karst. Foods.

[13] Saleh R.M., Awadin W.F. (2017). Biochemical and histopathological changes of subacute cadmium intoxication in male rats. Environ. Sci. Pollut. Res. Int..

[14] Das A., Joardar M., Chowdhury N.R., De A., Mridha D., Roychowdhury T. (2021). Arsenic toxicity in livestock growing in arsenic endemic and control sites of West Bengal: Risk for human and environment. Environ. Geochem. Health.

[15] Kumar A., Kumar A., Cabral-Pinto M., Chaturvedi A.K., Shabnam A.A., Subrahmanyam G., Mondal R., Gupta D.K., Malyan S.K., Kumar S.S. (2020). Lead Toxicity: Health hazards, influence on food chain, and sustainable remediation approaches. Int. J. Environ. Res. Public Health.

[16] Tumolo M., Ancona V., Paola D.D., Losacco D., Campanale C., Massarelli C., Uricchio V.F. (2020). Chromium Pollution in European Water, Sources, Health Risk, and Remediation Strategies: An Overview. Int. J. Environ. Res. Public Health.

[17] (2017). National Food Safety Standard Contamination Limit in Food.

[18] (2006). Setting Maximum Levels for Certain Contaminants in Foodstuffs.

[19] Peng S., Zhang H., Song D., Chen H., Lin X., Wang Y., Ji L. (2022). Distribution of antibiotic, heavy metals and antibiotic resistance genes in livestock and poultry feces from different scale of farms in Ningxia, China. J. Hazard. Mater..

[20] Korish M., Attia Y. (2020). Evaluation of heavy metal content in feed, litter, meat, meat products, liver, and table eggs of chickens. Animals.

[21] Yabe J., Nakayama S., Ikenaka Y., Muzandu K., Choongo K., Mainda G., Kabeta M., Ishizuka M., Umemura T. (2013). Metal distribution in tissues of free-range chickens near a lead-zinc mine in Kabwe, Zambia. Environ. Toxicol. Chem..

[22] TShah A.Q., Kazi T.G., Baig J.A., Afridi H.I., Kandhro G.A., Arain M.B., Kolachi N.F., Wadhwa S.K. (2010). Total mercury determination in different tissues of broiler chicken by using cloud point extraction and cold vapor atomic absorption spectrometry. Food Chem. Toxicol..

[23] Wang W., Zhang W., Zhang X., Yang Q., Zhu F. (2017). Tracing heavy metals in ‘swine manure-maggot-chicken’ production chain. Sci. Rep..

[24] Ping Z., Huiling Z., Wensheng S. (2009). Biotransfer of heavy metals along a soil-plant-insect-chicken food chain: Field study. J. Environ. Sci..

[25] (2010). Feeding Management Regulations of Yellow-Feathered Chicken.

[26] (2006). Quality Management Practice for Broiler Slaughtering.

[27] Ogbomida E., Nakayama S., Bortey-Sam N., Oroszlany B., Tongo I., Enuneku A., Ozekeke O., Ainerua M., Fasipe I., Ezemonye L. (2018). Accumulation patterns and risk assessment of metals and metalloid in muscle and offal of free-range chickens, cattle and goat in Benin City, Nigeria. Ecotoxicol. Environ. Saf..

[28] Paz M., Correia-Sá L., Vidal C., Becker H., Longhinotti E., Domingues V., Delerue-Matos C. (2017). Application of the QuEChERS method for the determination of organochlorine pesticide residues in Brazilian fruit pulps by GC-ECD. J. Environ. Sci. Health Part B.

[29] Pappas R., Polzin G., Zhang L., Watson C., Paschal D., Ashley D. (2006). Cadmium, lead, and thallium in mainstream tobacco smoke particulate. Food Chem. Toxicol..

[30] Hu Y., Zhang W., Chen G., Cheng H., Tao S. (2018). Public health risk of trace metals in fresh chicken meat products on the food markets of a major production region in southern China. Environ. Pollut..

[31] Elkribi-Boukhris S., M’Hamdi N., Boughattas I., Helaoui S., Banni M. (2022). Assessment of heavy metal pollution transfer and human exposure risks from the consumption of chicken grown in mining-surrounding areas. Environ. Sci. Pollut. Res..

[32] Kabeer M., Hameed I., Kashif S., Khan M., Raza S. (2021). Contamination of heavy metals in poultry eggs: A study presenting relation between heavy metals in feed intake and eggs. Arch. Environ. Occup. Health.

[33] Babatunde O.O., Jendza J.A., Ader P., Xue P., Adedokun S.A., Adeola O. (2020). Response of broiler chickens in the starter and finisher phases to 3 sources of microbial phytase. Poult. Sci..

[34] Ao T., Pierce J.L., Pescatore A.J., Cantor A.H., Dawson K.A., Ford M., Paul M. (2011). Effects of feeding different concentration and forms of zinc on the performance and tissue mineral status of broiler chicks. Br. Poult. Sci..

[35] He Y., Wang Y., Sun B., Li S., Jiang G., Sun X., Guo Y., Xing M. (2016). Simultaneous analysis 26 mineral element contents from highly consumed cultured chicken overexposed to arsenic trioxide by inductively coupled plasma mass spectrometry. Environ. Sci. Pollut. Res..

[36] Akramzadeh N., Ramezani Z., Ferdousi R., Akbari-Adergani B., Mohammadi A., Karimian-khosroshahi N., Famenin B., Pilevar Z., Hosseini H. (2020). Effect of chicken raw materials on physicochemical and microbiological properties of mechanically deboned chicken meat. Vet. Res. Forum.

[37] Lee J., Hwang J., Lee H., Kim T., Choi J., Gang G. (2019). Effects of food processing methods on migration of heavy metals to food. Appl. Biol. Chem..

[38] Iwegbue C.M., Overah C.L., Ebigwai J.K., Sarah O.N., Godwin E.N., Eguavoen O. (2011). Heavy metal contamination of some vegetables and spices in Nigeria. Int. J. Biol. Chem. Sci..

[39] Shim J., Cho T., Leem D. (2018). Heavy metals in spices commonly consumed in Republic of Korea, Food additives and contaminants, Part B. Surveillance.

[40] Fu X., Chen E., Ma B., Xu Y., Hao P., Zhang M., Ye Z., Yu X., Li C., Ji Q. (2021). Establishment of an indirect competitive enzyme-linked immunosorbent method for the detection of heavy metal cadmium in food packaging materials. Foods.

[41] Wang Y., Xiong C., Luo W., Li J., Tu Y., Zhao Y. (2021). Effects of packaging methods on quality of heavy metals-free preserved duck egg during storage. Poult. Sci..

